# Cardiovascular disease risk prediction in older people: a qualitative study

**DOI:** 10.3399/BJGP.2020.1038

**Published:** 2021-09-07

**Authors:** Denise Ann Taylor, Katharine Ann Wallis, Sione Feki, Sione Sengili Moala, Manusiu Latu, Elizabeth Fono Fanueli, Padmapriya Saravanakumar, Susan Wells

**Affiliations:** Faculty of Health, Victoria University of Wellington, Wellington, New Zealand.; Primary Care Clinical Unit, University of Queensland, Brisbane, Australia.; Auckland and Waitemata District Health Boards, Auckland, New Zealand.; South Auckland, New Zealand.; Faculty of Medical and Health Sciences, University of Auckland, Auckland, New Zealand.; Faculty of Medical and Health Sciences, University of Auckland, Auckland, New Zealand.; Centre for Active Ageing, Auckland University of Technology, Auckland, New Zealand.; Faculty of Medical and Health Sciences, University of Auckland, Auckland, New Zealand.

**Keywords:** ageing, caregivers, multimorbidity, primary health care, social support, social work

## Abstract

**Background:**

Despite cardiovascular disease (CVD) risk prediction equations becoming more widely available for people aged ≥75 years, views of older people on CVD risk assessment are unknown.

**Aim:**

To explore older people’s views on CVD risk prediction and its assessment.

**Design and setting:**

Qualitative study of community-dwelling older people in New Zealand.

**Method:**

A diverse group of older people was purposively recruited. Semi-structured interviews and focus groups were conducted, transcribed verbatim, and thematically analysed.

**Results:**

Thirty-nine participants (mean age 74 years) of Māori, Pacific, South Asian, and European ethnicities participated in one of 26 interviews or one of three focus groups. Three key themes emerged: poor knowledge and understanding of CVD and its risk assessment; acceptability and perceived benefit of knowing and receiving advice on managing personal CVD risk; and distinguishing between CVD outcomes — stroke and heart attack are not the same. Most participants did not understand CVD terms, but were familiar with the terms ‘heart attack’ and ‘stroke’, and understood lifestyle risk factors for these events. Participants valued CVD outcomes differently, fearing stroke and disability — which might adversely affect independence and quality of life — but were less concerned about a heart attack, which was perceived as causing less disability or swifter death. These findings and preferences were similar across ethnic groups. All but two participants wanted to know their CVD risk, how to manage it, and distinguish between CVD outcomes. Those who did not wish to know perceived this as something only their God could decide.

**Conclusion:**

To inform clinical decision making for older people, consideration of an individual’s wish to know their risk is important, and risk prediction tools should provide separate event types rather than just composite outcomes.

## INTRODUCTION

Cardiovascular disease (CVD) is a leading cause of health loss and mortality in older people.^1^ In terms of preventive care, there is evidence that reducing smoking,^2^ blood pressure (BP),^3^ and lipids^4^ are associated with improved CVD outcomes for adults at any age, with the benefits largely determined by patients’ pre-treatment CVD risk (using 5-year or 10-year risk equations as key prognostic tools). CVD risk prediction equations are available for older people including:

- the UK QRISK3 for people aged 25–84 years;^5^
- the US Pooled Cohort Equations for people aged 40–79 years;^6^
- the European Systematic COronary Risk Evaluation in older people (SCORE O.P.) for people aged 65–80 years;^7^ and
- the Canadian CVD Population Risk Tool (CVDPoRT) for people aged 20–105 years.^8^

In New Zealand, there are new primary prevention CVD risk equations for people aged 30–74 years,^9^ but no specific equations as yet for those aged ≥75 years. National guidelines recommend that healthy older people with few comorbidities and a life expectancy ≥5 years have their CVD risk assessed and managed in the same way as younger people, and that risk management is at the discretion of the clinician, taking into consideration CVD risk, potential benefits and risks of treatment, and patient preferences.^9^^,^^10^

Qualitative studies were found examining the preferences for discussing prognostic information in older adults with late-life disability,^11^ those with heart failure,^12^^,^^13^ and those with cancer, chronic obstructive pulmonary disease, or other chronic disease.^13^^–^^15^ The recurring theme was that older people wanted to discuss their prognosis, to be prepared, to anticipate need for treatment or potential consequences, and to plan ahead.^11^^–^^16^ However, no studies could be found that investigated older people’s views about CVD risk assessment, whether a risk prediction estimate would be of value to them, or whether they would want to know their CVD risk, the outcomes they would want to avoid (for example, death and/or hospitalisation, stroke and/or heart attack), and whether preferences for CVD prognosis might vary, for example, across ethnicity groups or sex.

This study aimed to explore the views of a diverse group of older people in New Zealand regarding CVD risk prediction and its assessment.

**Table:** How this fits in

Cardiovascular disease (CVD) risk prediction equations are increasingly available for people aged ≥75 years, and predict a combined set of outcomes. The views of older people on CVD risk assessment were explored and their preferences regarding prediction of different CVD outcomes. The findings suggest older people want to know their CVD risk and how to manage this, but they distinguish between CVD outcomes — fearing a stroke and being less concerned about a heart attack. Developers of risk prediction tools should consider both combined CVD outcomes and provide separate estimates for future coronary and stroke events. Findings highlight that most older people want to know their CVD risk but distinguish between CVD outcomes. This new knowledge may be used by GPs to inform discussions and shared decision making about CVD risk management in older people.

## METHOD

### Design and setting

A descriptive qualitative methodological approach using focus group and semi-structured interview methods was employed, with an inductive and iterative thematic analysis stance.^17^ Potential participants were community-dwelling older people in New Zealand aged ≥75 years for European and ≥65 years from four ethnic groups: Māori, Pacific (including Tongan, Samoan, Niuean, Cook Islands), South Asian, and European ethnicities. These four ethnic groups were chosen because the majority of older people in New Zealand claim European ethnicity; and Māori (indigenous people of New Zealand), Pacific, and South Asian groups experience CVD events on average 10 years earlier than European New Zealanders.^18^^,^^19^

### Recruitment

Budgetary constraints influenced the sample size and up to six older people from each ethnic group were purposefully recruited, although interested participants were not turned away. As this was a small study it did not aim for data saturation; a diverse range of rich data was expected owing to the heterogeneity of the participants.

Participants were recruited using flyers at local libraries, social groups such as University of the Third Age (UoTA), places of worship, other community groups, three general practice clinics, and by word of mouth. At two UoTA events, a non-CVD presentation was given before introducing the project, so for some participants a relationship was established before participation. Participant information sheets outlined details of the research and researchers, and invited potential participants to take part in a single one-toone interview with the researcher at a place of their choice, or as part of one focus group with people from the same ethnic group.

### Data collection

A topic guide was developed (Supplementary Appendix S1) asking participants what they understood by the term CVD, if they were aware that the risk of experiencing CVD was predictable (CVD risk assessment), whether they would want to know their risk, what CVD outcomes were of most concern to them, and their preferences regarding CVD prediction. The topic guide was piloted with male and female older people, and the use of medical jargon was adjusted accordingly. All interviews and focus groups were digitally recorded, translated as appropriate, transcribed verbatim, and written up as a de-identified transcript for participants to comment on. Twelve participants commented positively on the accuracy of transcribing and maintenance of confidentiality. Tongan elders were member checked, receiving an overview of their findings. They and other participants await this publication, which will be shared with all those requesting it.

### Cultural considerations

Ethnic-specific researchers were employed to help with recruitment, data collection, interpretation, and analysis in populations where the level of spoken English was poor. This ensured that analytical processes captured cultural and social nuances, and findings were embedded within specific cultural contexts. For example, two authors highlighted in the analyses the deep respect Pacific peoples had for medical practitioners, but which was entirely different from their devotion to their God. Authors worked in partnership with Tongan elders to arrange separate sex focus groups to enable female participants to speak freely. Refreshments were provided to participants as an expression of creating a safe place to share stories and demonstrate generosity.^20^ Talanoa, a Pacific research methodology, was used to guide the interviews and focus groups to foster a culturally safe environment that enabled participants to feel comfortable in sharing their experiences.^21^ A Mātauranga Māori approach was used,^22^ guided by a lead Māori researcher, which supported key Māori values of tikanga (cultural principles guiding appropriateness of action and behaviour) and kawa (cultural practices) to be embedded throughout the project to honour sharing of knowledge, the mana (essence) of the person sharing it, and the protection of knowledge (kaitiakitanga).^23^

### The research team

The lead researcher was an experienced qualitative researcher working in older people’s health, and was responsible for recruitment, data collection, analysis, and delivery of project objectives. The lead researcher completed the European cohort interviews, most of the South Asian (*n* = 5/6) and Māori (*n* = 6/7) interviews, and facilitated one focus group in English for Tongan elders. Two authors conducted further interviews for Pacific, Māori, and South Asian participants. Two other authors (Tongan investigators) led two focus groups (one with male and one with female participants) in Old Tongan, which were translated and transcribed by a separate author, with verification by the authors who led the focus groups. Field notes were captured after each interview or focus group to contextualise the shared information and to add new questions to the topic guide as appropriate.

There were unique analysis teams for each cohort (European, Pacific, South Asian, and Māori). Each analysis team used personal reflection and cultural and contextual guidance from each other to ensure findings were grounded in the reality of the participant and their community. Potential participants opted into the study, and two people who expressed an interest in the study were unable to attend owing to CVD-related hospitalisation. Owing to the data recruitment strategy, however, it was unclear how many potential participants declined to participate.

### Analysis

Each participant was assigned an identification code for the analysis according to their ethnicity, sex, and participant number in that ethnic group. (For example, the first Māori male participant is identified as M-M1 and the third South Asian male is SA-M3.) An iterative inductive approach to thematic analysis was used,^17^ owing to its flexibility in relation to the data it is applied to and because it is not tied to one epistemological stance. To honour the cultural and social perspectives of each ethnic group, each group was analysed separately followed by an overarching analysis. Steps included:

- familiarisation with the data;
- generation and refinement of codes; and
- searching for themes that were then named and refined.^17^

To ensure reliability and validity, at least two members of the team reviewed all data, codes, and themes, and agreed coding and naming decisions at each of the four coding iterations before progressing. This validity and reliability were enhanced by ethnic-specific researchers who contextualised findings, the use of quotations to demonstrate key findings and honour participant stories, and the consolidated criteria for reporting qualitative research (COREQ) to guide reporting.^24^ A data management tool was not used to support coding to allow an opportunity to debate the analytical process in real time with the whole research team until consensus was achieved.

## RESULTS

Table 1 describes the 39 participants aged 61–91 years (mean age 74 years; *n* = 19 female, *n* = 20 male) who were recruited from four ethnic groups: Māori (*n* = 7), Pacific (*n* = 15), South Asian (*n* = 8), and European (*n* = 9). All but two participants had ≥1 of the following diagnoses: diabetes, hypertension, heart failure, atrial fibrillation, angina, or history of coronary bypass or stent surgery.

**Table 1.** Participant characteristics and method of data collection

**Ethnic group**	**Total, *n***	**Female, *n***	**Average age, years**	**Range, years**	**Interview, *n***	**Focus groups, *n***
NZ European	9	5	81.0	73–91	9	—
Māori	7	2	69.6	65–76	7	—
Pacific	15	10	72.2	61–88	4^a^	11^b^
South Asian	8	2	72.3	65–83	8^c^	—

a n *= 2 Samoan,* n *= 1 Niuean, and* n *= 1 Cook Islands.*

b *All participants were Tongan.*

c *Two interviews included both husband and wife.*

Participants took part in one of 26 interviews or one of three focus groups. Interviews ranged from 25–60 min, and focus groups from 20–40 min. Most interviews were conducted in the participant’s home; one was conducted in a church, two at participants’ workplaces, and two at participants’ general practice clinic. Three South Asian participants had a non-participating member of their family present at the interview for support, and two South Asian couples were interviewed together as husband and wife. Three focus groups were conducted with Tongan elders in their weekly meeting space. Two focus groups were conducted in Old Tongan (one with four male elders, and the other with five female elders [two were accompanied by their niece]), and one in English (with two female elders).

A series of iterative coding and recoding resulted in three superordinate themes, each with three or four subthemes. These are presented in Box 1.

**Box 1.** Hierarchical coding framework

**1.**	**Poor knowledge and understanding of CVD and its risk assessment** 1.1 I don’t know what it (CVD) means 1.2 How do I avoid a risk of stroke or heart attack? 1.3 My genetics mean I won’t get a heart attack 1.4 Will risks of medication outweigh any benefits?
**2.**	**Acceptability and perceived benefit of knowing and receiving advice on managing personal CVD risk** 2.1 We need to know before anything happens 2.2 I do not want to know 2.3 I would do anything to reduce risk 2.4 If I knew I was high risk I would do more about it
**3.**	**Distinguishing between CVD outcomes — stroke and heart attack are not the same** 3.1 I would prefer to have a heart attack 3.2 I want quality of life, not dependence 3.3 I don’t want to die of any

*CVD = cardiovascular disease.*

### Theme 1: Poor knowledge and understanding of CVD and its risk assessment

Few participants recognised or understood the term ‘cardiovascular disease’, although most knew what ‘having a heart attack or stroke’ meant. Some participants were aware that the risk of experiencing a CVD event was predictable, but the majority were not. Many Tongan participants were shocked to discover that CVD risk could be predicted and managed, reporting that they would have liked to have known this when they were younger so they could have done something about it. Other Pacific participants thought a diagnosis of CVD was akin to a death sentence, while all South Asian participants thought it was inevitable. Many participants believed that their own health would follow that of their own family history, for example, that if their mother had had a stroke then they would also have a stroke. See Box 2 for exemplar quotes.

**Box 2.** Superordinate code 1 themes and example quotations

**Subthemes**	**1. Poor knowledge and understanding of CVD and its risk assessment**
1.1. I don’t know what it (CVD) means	*‘Not really, but I know if there’s a problem with the heart they call it cardio. That’s how they tackle everything like that. But I don’t know much about it.’* (SA-M2) *‘The red blood cells which supplies heart, supplies blood to the heart, that is, that is what gets affected, isn’t it, in general terms?’* (E-F3)
1.2. How do I avoid a risk of stroke or heart attack?	*‘Am I likely to get a stroke or a heart attack or any form of cardiovascular disease? And if so, what can I do to change my lifestyle to minimise those risks?’* (SA-M6) *‘What are the habits that would lead to a heart attack? What health conditions would lead to a heart attack, and what they should do to avoid getting in that situation?’* (E-F2)
1.3. My genetics mean I won’t get a heart attack	*‘I’m pretty sure that I won’t get the heart attack because my genetic is on my mother. She died of the kidney failure and my problem is the same.’* (SA-M2)
1.4. Will risks of medication outweigh any benefits?	*‘* [On CVD risk] *Well not as much as I would like in the sense that, I think what I’ve been given is in very broad brush kind of terms. And even a bald statement of “You’ve got a 15 percent chance, or whatever it is, of being admitted in the next five years with heart or a stroke.” I mean OK it’s a sort of risk and it’s worth taking, yeah and I’d rather know that than not know it. But it’s not all that helpful in the things that I’m really concerned about. Is what am I doing that can help this and what are the risks of doing that? And is it worth taking some risky thing for some pretty marginal kind of benefit? And so that’s where I don’t actually think the information has been as robust as I would like, because as I’ve said I’m not sure that it’s there for people of my age and my range of comorbidities.’* (E-M4)

*CVD = cardiovascular disease. E = European. F = female. M = male. SA = South Asian.*

### Theme 2: Acceptability and perceived benefit of knowing and receiving advice on managing personal CVD risk

Most (*n* = 37/39) participants wanted to know their CVD risk, although some believed that predictions about the future were in the hands of God and not of their GP. Most participants wanted to know what they could do to reduce their CVD risk, and were aware of CVD lifestyle recommendations concerning diet, exercise, smoking, and alcohol, as well as CVD preventive medications. The Tongan elders said they participated in the study to learn about CVD so they could share this new knowledge with their children and grandchildren to empower better health and lifestyle choices. Some participants accessed information via the internet, but many wanted information in a form that could be shared with family either verbally or through a printed document. See Box 3 for exemplar quotes.

**Box 3.** Superordinate code 2 themes and example quotations

**Subthemes**	**2. Acceptability and perceived benefit of knowing and receiving advice on managing personal CVD risk**
2.1. We need to know before anything happens	*‘I think this is really important these things, to know beforehand and then we can be cautious and prevent these things from happening. But what I believe is it will be useful for the future, when our children grow up and learn and become knowledgeable.’* (T-M3, FG1) *‘People should know the risk before. After a certain age you are prone to so many things and it is better for them to know exactly where they stand. At least they can change their lifestyle … If I’m having a high risk then I would like to know what I can do to avoid that, yeah, definitely.’* (SA-M4)
2.2. I do not want to know	*‘No, I would not want to know from our GP. I just leave my life to our maker, that’s the reason I don’t want my GP to say to me, “You’re going to have a heart attack, in two to three years’ time.” That’s like a predicting, my days but it’s not him … it’s our Lord, that’s my belief anyway.’* (N-F1) *‘If it was offered and somebody had the choice to accept that, then that would be OK. But just to tell somebody that there’s this prediction you will have a heart attack, or some form of heart problem in the next five years, would be a bit much for some people to take I’m sure.’* (E-F5)
2.3. I would do anything to reduce risk	*‘I would like to be told before something happened. I’d like the doctor say, “Well we’ve checked your blood test and there’s something wrong with your heart” …* [I would] *do anything the doctor wanted me to do, you know? I don’t know what, if they put you on pills.’* (M-M4)
2.4. If I knew I was high risk I would do more about it	*‘I understand that it’s not probably exact science, it’s not going to say, “Well you’ve got three and a half years before you have a stroke, or four years before”, it’s just a general situation … if I knew my risk was high, I would take more notice of the symptoms.’* (M-M1)

*E = European. F = female. FG = focus group. M = Maori/male. N = Niuean. SA = South Asian. T = Tongan.*

### Theme 3: Distinguishing between CVD outcomes — stroke and heart attack are not the same

Participants perceived stroke as a separate disease unrelated to heart disease. They feared strokes, which were associated with prolonged hospitalisation adversely affecting their quality of life and their ability to think and communicate, as well as being a burden to the rest of the family. They were less concerned about heart attacks, believing they could be treated (for example, with stents) and caused less disability or a swifter death. Participants believed that the risk of stroke and heart attack were different because a stroke was much more devastating in terms of personal outcomes and potential impact on family. Consistent with this understanding, most participants believed that risk for the different CVD outcomes should be predicted separately. These preferences regarding CVD risk prediction and outcomes were consistent across participants, regardless of ethnicity. See Box 4 for exemplar quotes.

**Box 4.** Superordinate code 3 themes and example quotations

**Subthemes**	**3. Distinguishing between CVD outcomes — stroke and heart attack are not the same**
3.1. I would prefer to have a heart attack	*‘I think a heart attack is different. Just a heart attack where you may damage a portion of your heart during the heart attack, I think I could cope with that. As long as I wasn’t impeded in my ability to get around and enjoy life.’* (E-F5) *‘I wouldn’t want to be dependent on anyone, whereas a heart attack, OK, you may have a severe heart attack and you’re gone, that’s OK.’* (SA-F2)
3.2. I want quality of life, not dependence	*‘I’ve immediately got some reservation about lumping those two together. Because being hospitalised for a heart attack is different to me from being hospitalised for a stroke. My mental function is important to me in my old age and I don’t want a heart attack either, but I’m conscious that a lot of heart attacks these days can be ably managed with stenting and various other things … but those two that have been lumped together are different risks for me in terms of how they would affect me and what I can do in my old age.’* (E-M4) *‘Given the option between stroke, cardiac disease, and death, I think that would be the best* [heart attack]. *Because after a stroke, life is not really, the quality of life is not the best.’* (SA-M6)
3.3. I don’t want to die of any	*‘The death probably not, no. I think from what I’ve seen, a stroke would worry me more than anything. The fact of being very active, right throughout my life, brought up on a farm, and carried on since then. Played a lot of sport, to be an active mind in a body that is not going to respond and give me the freedom and the movement and so on would worry me more than, I think, potentially a heart attack. And I would expect that maybe I can do more to prevent a heart attack, maybe, than a stroke, I don’t know, but that’s just my, you know, layman’s view on it.’* (M-M3) *‘For me, I would choose not to have all of them.’* (T-M4, FG1) *‘Prefer to prevent a stroke, don’t particularly want to die from either, but I realise that death is becoming closer. And so I don’t really wanna be disabled and unable to walk or, you know, all these other things. And so, I’m not afraid of death, but I prefer not to die. I’m enjoying life, I regard myself as well despite all these ailments.’* (E-M4)

*E = European. F = female. FG = focus group. M = Maori/male. SA = South Asian. T = Tongan.*

## DISCUSSION

### Summary

This study explored the views of a diverse group of 39 older people about CVD and its risk prediction. Most participants had CVD or CVD risk factors, and many were taking CVD preventive medications. Findings suggest that older people held poor understanding of the term ‘cardiovascular disease’ and its risk assessment/prediction, but knew what having a ‘heart attack’ or ‘stroke’ meant, and were aware of lifestyle risk factors for CVD. Most participants reported wanting to know their CVD risk and how to reduce it, but two would rather leave such predictions to their God. Importantly, participants distinguished between CVD outcomes, fearing a stroke due to perceived disability and effect on independence and quality of life, but being less concerned about a heart attack, which was perceived to be treatable and cause less disability or swifter death.

### Strengths and limitations

To the authors’ knowledge, this study is the first to investigate elders’ preferences regarding CVD risk prediction. A strength of the study is the inclusion of an ethnically diverse group of older people of Māori, Pacific, South Asian, and European ethnic groups, from different geographical sites in New Zealand including city, rural, and urban. The research team included ethnic-specific researchers to contextualise findings and sense-check data within specific ethnic groups.

Embedded within the methodologies were key processes to ensure the trustworthiness of the findings. These included collaboration with ethnic-specific researchers to ensure credibility, dependability, and contextualisation of data, and reflexivity of each researcher when engaged with coding reiterations, so that the stories of the participants, and not the researchers, came through. Qualitative data may not be completely transferable, but, given the convergence of views, the findings are likely to be reflected in similar population groups.

Important limitations are that participants were a small sample of self-selected volunteers living at home or with extended family, or in retirement complexes. Therefore, they may not be representative of all older people, in particular of those in aged residential care. Furthermore, this was a small study that did not aim for data saturation, as a diverse range of rich data was expected owing to the heterogeneity of the participants. However, for the question on the views of older people on CVD risk prediction, data saturation was reached with only three opinions; the majority wanting to know their risk so they could lessen it, that stroke risk was more important than coronary, and the belief that the only person who should deliver such news was their God.

### Comparison with existing literature

It is not surprising that participants did not understand the term ‘cardiovascular disease’ because it is a medical term for various diagnoses (for example, coronary heart disease [CHD], stroke, peripheral vascular disease, and heart failure) owing to arterial atherosclerosis. While CVD medical management seeks to mitigate the pathophysiological impact of arterial atherosclerosis, the benefit and harms of treatment are usually conveyed to patients by clinicians according to risk factor (for example, reduce BP or cholesterol) or common CVD outcomes (for example, reduce risk of a heart attack or stroke).^18^

However, it is perhaps surprising that participants were unaware that CVD risk could be predicted and managed, given that many were on CVD preventive medications. This may be in part because, in New Zealand, the Ministry of Health has promoted CVD risk assessment as having a ‘heart and diabetes check’ and these checks are recommended for people aged <75 years.^10^^,^^18^ With the exception of two participants, there was substantial interest in the fact that the risk of CVD outcomes could be predicted, as well as a desire to be offered and know their own prognosis, and to discuss and understand it.

This study’s findings are consistent with other studies in that the majority of participants are reported to be interested in their prognosis or individualised survival statistics.^11^^–^^16^^,^^19^ For example, in a study of 40 older Americans (African American, Chinese American, European American, Latinos, and other) 75% indicated they would want to discuss prognosis with their doctor to prepare logistically/financially, emotionally, or spiritually, as well as to involve family and friends, make health-related decisions, and make the most of the time they have left.^11^ However, as in the present study, some did not. Indeed, one in four participants said they would prefer not to discuss prognosis as they thought the information was not useful, was too emotionally distressing, or that doctors cannot estimate prognosis (only God can).^11^ Furthermore, similar to the present study, a sense of helplessness stemming from a family history (for example, ‘my mother had a stroke and therefore so will I’) was also expressed. In a study of older Chinese females on health and cancer screening,^25^ the authors report themes of genetic predisposition (for example, inheritance from their ancestors) and a sense of fatalism towards illness (what will happen will happen).

Earlier CVD risk equations have been developed for separate categories of CVD outcomes. For example, in 1991 Anderson *et al* published separate equations for myocardial infarction, CHD, death from CHD, stroke, CVD, and death from CVD.^26^ However, more recent equations that include older patients have comprised only one composite outcome.^5^^–^^8^ The present findings suggest that, for older people, it is important for CVD risk prediction tools to not only identify the magnitude of CVD risk, but also to separate outcomes such as non-fatal stroke, non-fatal CHD, fatal CVD, and all-cause mortality. These separate prognostic outcomes are important for discussions and decision making regarding the potential benefits and harms of treatment, especially when the potential harms (adverse effects) may be experienced immediately, while the potential benefits may only be reaped after many years. The present study is also consistent with national guidance^10^ and findings made by Jansen *et al*,^27^ which suggest participants want to know their prognosis and be involved in clinical discussions and decisions.^28^

### Implications for research and practice

All but two participants wanted to know their CVD risk and how to manage it, and welcomed individualised clinician advice. However, because they distinguish between CVD outcomes such as stroke and myocardial infarction, CVD risk prediction algorithms should be developed to provide separate prognostic indicators for the separate CVD outcomes, taking into consideration both the magnitude of CVD risk and the type of CVD outcome. Importantly, participants in this study valued interaction with their GP and trusted them to make the best decision for them as an individual. A recent systematic review of 47 clinical practice guidelines on CVD prevention found that, although older people are mentioned in most guidelines, the information provided to guide treatment for older people is vague and limited.^28^ Clearer guidance is needed for tailoring management to each older person’s context and facilitating greater involvement in shared decision making that considers patient preferences and goals.^18^
